# Stable Disease Achieved with Sequential Immunochemotherapy and Anti-Angiogenic TKI in Recurrent Metastatic Hidradenocarcinoma: A Case Report and Literature Review

**DOI:** 10.32604/or.2026.080462

**Published:** 2026-07-16

**Authors:** Shidi Wen, Lu Wang, Ying Jiang, Zhiyang Zhang, Yuejuan Cheng

**Affiliations:** 1 Clinical Medicine College, Chinese Academy of Medical Sciences & Peking Union Medical College, Beijing, China; 2 Department of Medical Oncology, National Cancer Center/Cancer Hospital, Chinese Academy of Medical Sciences & Peking Union Medical College, Beijing, China; 3 Department of Pathology, Peking Union Medical College Hospital, Chinese Academy of Medical Sciences & Peking Union Medical College, Beijing, China; 4 Department of Medical Oncology, Peking Union Medical College Hospital, Chinese Academy of Medical Sciences & Peking Union Medical College, Beijing, China

**Keywords:** Hidradenocarcinoma, sweat gland carcinoma, immunotherapy, extramammary Paget disease, case report

## Abstract

**Background:** Hidradenocarcinoma is a rare and highly aggressive malignancy with limited therapeutic options. This report describes the clinical course and treatment response of a patient with recurrent metastatic hidradenocarcinoma treated with sequential immunochemotherapy combined with anti-angiogenic therapy, with the aim of providing further insight into potential treatment strategies for this rare malignancy. **Case Description:** A 60-year-old male initially presented in 2020 with scrotal erythema and was diagnosed with hidradenocarcinoma after surgery. Despite surgical treatment, he developed recurrent disease with diffuse metastases. First-line chemoimmunotherapy (sintilimab, cisplatin, 5-fluorouracil; six cycles) achieved a progression-free survival (PFS) of 6 months. Following disease progression, second-line therapy (toripalimab, nab-paclitaxel, anlotinib; eight cycles) was administered, resulting in sustained stable disease with a subsequent PFS of 8 months. Radiotherapy was used for brain metastases. The total follow-up duration exceeded 4 years until the patient was lost to follow-up in December 2024. **Conclusions**: This case suggests that sequential programmed death-1 (PD-1) blockade-based immunochemotherapy combined with anti-angiogenic therapy may provide clinically meaningful disease control in metastatic hidradenocarcinoma, even in the setting of low programmed death-ligand 1 (PD-L1) expression and microsatellite stability. Our findings support a potential role for immunotherapy in sweat gland carcinomas and highlight the importance of individualized multimodal treatment strategies for this rare malignancy. Further studies are needed to identify predictive biomarkers and establish optimal therapeutic approaches.

## 1 Introduction

Hidradenocarcinoma is a rare but aggressive adnexal malignancy with sweat gland differentiation, with an estimated incidence of 0.45 per million person-years [1]. The disease is associated with a high risk of metastasis and a 10-year overall survival of approximately 60% [2]. Population-based studies indicate that hidradenocarcinoma most commonly arises in the head and neck region, followed by the trunk and extremities [3]. Biologically, it shares features with extramammary Paget disease (EMPD), which typically occurs in apocrine-rich areas such as the genital or axillary skin [4,5]. In some cases, EMPD may coexist with or represent a secondary manifestation of underlying adnexal carcinomas, including hidradenocarcinoma [6]. These overlapping clinical and histological features can complicate diagnosis and management.

Surgical excision remains the primary treatment for localized hidradenocarcinoma [7,8]. However, no standard systemic therapy exists for metastatic and recurrent disease, largely due to the rarity of the condition and the lack of large-scale studies. Radiotherapy and chemotherapy, including regimens based on 5-fluorouracil or capecitabine, have been reported to provide local disease control in selected reports [9]. Additional treatment approaches, such as targeted therapy, hormonal therapy, and electrochemical therapy, have also been described in isolated reports [9]. Given the limited evidence base, organizing clinical trials is challenging, and these individual experiences may offer valuable insights into potential therapeutic strategies.

Immunotherapy has recently transformed the treatment landscape for several malignancies, particularly those with high tumor mutational burden (TMB) or evidence of immune infiltration. Combination regimens involving dual immune checkpoint inhibitors (ICIs) or ICIs with chemotherapy have demonstrated efficacy in aggressive cancers such as melanoma and non-small cell lung cancer [10,11,12,13]. However, the role of immunotherapy in hidradenocarcinoma remains underexplored, with only a few case reports documenting its efficacy and safety. This limited evidence underscores the need to explore novel therapeutic approaches for this rare disease.

Here, we present a patient with histologically diagnosed hidradenocarcinoma who developed postoperative recurrence and distant metastases. The patient was treated with sequential immunochemotherapy as both first- and second-line therapy, achieving prolonged disease control. The aim of this report is to describe the clinical course and treatment response of metastatic hidradenocarcinoma treated with sequential immunochemotherapy, thereby providing further insight into potential treatment strategies for this rare malignancy. This case highlights the potential of immunochemotherapy as a treatment option for advanced hidradenocarcinoma and underscores the need for further research to establish standardized management strategies.

The requirement for Institutional Review Board (IRB) approval was waived by the IRB of Peking Union Medical College Hospital due to the retrospective nature of this case report. The handwritten informed consent was obtained from the patient. Besides, this study was prepared according to the CARE case report guideline, and a CARE checklist was provided. Please see Supplementary Material S1 for more details.

## 2 Case Presentation

**Figure 1:**
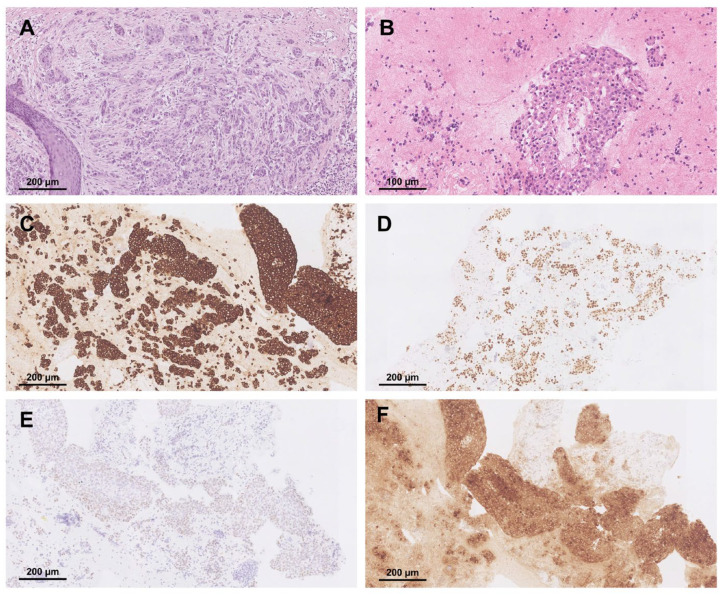
**Histopathological and immunohistochemical features of metastatic hidradenocarcinoma.** (**A**) Hematoxylin and eosin (H&E) staining of the scrotal skin lesion demonstrates dermal infiltration by adenocarcinoma. Tumor cells are arranged in glandular structures and exhibit abundant eosinophilic cytoplasm (original magnification ×100). (**B**) H&E staining of a fine-needle aspiration smear from an inguinal lymph node confirms the presence of metastatic adenocarcinoma cells (original magnification ×200). (**C**–**F**) Immunohistochemical staining of the metastatic inguinal lymph node lesion shows that the adenocarcinoma cells are positive for (**C**) pan-cytokeratin (AE1/AE3), (**D**) androgen receptor (AR), (**E**) GATA3, and (**F**) gross cystic disease fluid protein 15 (GCDFP-15) (original magnification ×100).

A 60-year-old man with a smoking history and no significant past medical history presented to Peking Union Medical College Hospital in 2020 with erythema of the right scrotum skin. Over several months, the lesion progressed to a 3-mm reddish nodule. He subsequently underwent excision of the nodule and wide local excision of the scrotal and penile skin with a 2-cm clinical margin. Histopathological examination confirmed hidradenocarcinoma and Paget’s disease, and surgical margins were tumor-free. In September 2022, the patient developed scrotal and penile edema accompanied by inguinal lymphadenopathy. On 16 January 2023, positron emission tomography–computed tomography (PET‒CT) revealed hypermetabolic foci in the subcutaneous tissue of the penile and scrotal regions, along with multiple enlarged lymph nodes in the retroperitoneum, pelvis, axilla, neck, and hilum, suggestive of metastatic spread. Biopsy of the scrotal skin and right inguinal lymph node performed on 21 February 2023, confirmed recurrence and metastasis. Microscopic examination of the skin lesion showed dermal infiltration by adenocarcinoma, with tumor cells arranged in glandular structures and exhibiting abundant eosinophilic cytoplasm (Fig. 1A). Fine-needle aspiration cytology of the inguinal lymph node confirmed metastatic adenocarcinoma (Fig. 1B). Immunohistochemical analysis demonstrated positive programmed death-ligand 1 (PD-L1) expression (clone 22C3; combined positive score [CPS] = 5) and microsatellite stable (MSS) status. Tumor cells were positive for pan-cytokeratin (AE1/AE3) (Fig. 1C), androgen receptor (AR) (Fig. 1D), focal GATA3 (Fig. 1E), gross cystic disease fluid protein 15 (GCDFP-15) (Fig. 1F), human epidermal growth factor receptor 2 (HER2; 1+), focal mammaglobin, and focal P63 (image not shown). S-100 staining was negative. Subsequent imaging was performed to assess distant disease. On 8 March 2023, pelvic contrast-enhanced magnetic resonance imaging (MRI) demonstrated multiple abnormal enhancing nodules in the lumbosacral spine and right acetabulum, raising suspicion for bone metastases. Radionuclide bone imaging performed on 26 May 2023, confirmed metastatic involvement of the lumbar spine and sacroiliac joint.

**Figure 2:**
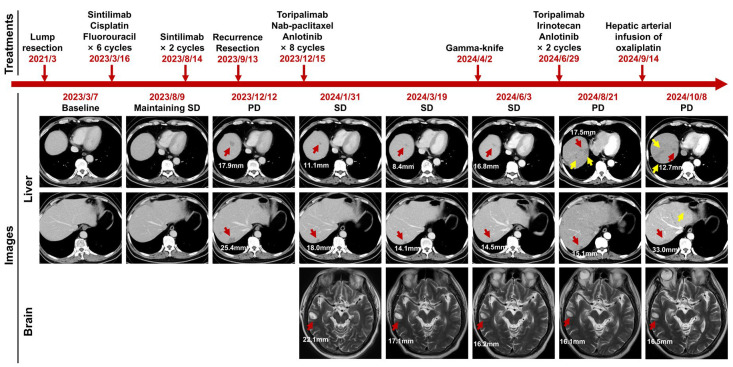
**Treatment timeline and therapeutic efficacy of the patient.** The upper panel illustrates the treatment timeline, including systemic therapies, radiotherapy, and major clinical events. The lower panel displays representative axial contrast-enhanced computed tomography (CT) images of the liver (upper row) and T1-weighted magnetic resonance imaging (MRI) of the brain (lower row) at key time points during the disease course. Tumor response was assessed according to Response Evaluation Criteria in Solid Tumors (RECIST) version 1.1. Red arrows indicate target lesions. Yellow arrows indicate new lesions that emerged during therapy.

The patient was diagnosed with metastatic advanced hidradenocarcinoma. In the absence of an established standard chemotherapy regimen, and after thorough discussion with the patient, combination immunotherapy was initiated based on institutional experience. On 16 March 2023, treatment began with sintilimab (200 mg intravenously [IV] on day 1), cisplatin (49 mg IV on days 1–3), and 5-fluorouracil (4.8 g via continuous IV infusion over 46 h, followed by 3 g over 60 h) (Fig. 2). Tumor response was assessed by contrast-enhanced computed tomography (CT) every two cycles (approximately every 6 weeks) according to Response Evaluation Criteria in Solid Tumors (RECIST) version 1.1 guidelines [14]. Zoledronic acid (4 mg) was administered concurrently to reduce the risk of skeletal-related events associated with bone metastases. After six cycles, the patient achieved stable disease (SD). The regimen was well-tolerated, with no adverse events of Grade 1 or higher according to Common Terminology Criteria for Adverse Events (CTCAE) version 5.0. Unfortunately, during maintenance therapy with sintilimab monotherapy, disease progression was observed in September 2023 with a perineal recurrence. Despite pre-existing bone and lymph node metastases, a second surgical resection was performed at another hospital to relieve local symptoms, including pain and swelling, following shared decision-making. Immunohistochemical analysis of the recurrent tumor showed persistent PD-L1 positivity (CPS = 3) and MSS status. Further molecular profiling identified mutations in the *BCL2L11* and *FOXA1* genes.

Following the detection of new metastatic lesions in the liver, skull, and cervical and thoracic vertebrae, second-line therapy was initiated on 15 December 2023, consisting of toripalimab (240 mg IV on day 1), nab-paclitaxel (200 mg IV on days 1 & 8), and anlotinib (10 mg orally on days 1–14). Tumor assessment after two and four cycles demonstrated SD. Notably, hepatic metastases decreased in size from 17.9 mm and 25.4 mm to 11.1 mm and 18.0 mm, and further to 8.4 mm and 14.1 mm, indicating meaningful antitumor activity. On 2 April 2024, the patient underwent Gamma Knife radiosurgery for brain metastases. After seven cycles of systemic therapy, surveillance imaging showed enlargement of a pre-existing hepatic lesion from 8.4 mm to 16.8 mm (Fig. 2); however, the overall response remained SD according to RECIST 1.1 criteria. Treatment was generally well-tolerated during the first seven cycles, with no immune-related adverse events or other adverse events ≥ Grade 1 observed. On 19 June 2024, following the first day of the eighth cycle, the patient developed anemia (hemoglobin 89 g/L; CTCAE Grade 2) and hypokalemia (serum potassium 2.9 mmol/L; CTCAE Grade 3), which were considered more likely related to chemotherapy than immunotherapy. No associated symptoms such as fatigue or nausea were reported. Immunochemotherapy was suspended, and supportive care with erythropoiesis-stimulating agents, iron, and potassium supplementation was initiated.

These toxicities prompted modification of the chemotherapy regimen while toripalimab and anlotinib were continued. On 29 June 2024, treatment was switched to toripalimab (240 mg IV on day 1), irinotecan (180 mg IV on days 1 & 8), and anlotinib (10 mg orally on days 1–14). Unfortunately, follow-up imaging on 21 August 2024, revealed multiple new liver metastases, consistent with progressive disease (PD), leading to discontinuation of this regimen. The patient subsequently received three cycles of hepatic arterial infusion chemotherapy with oxaliplatin; however, disease progression persisted. Imaging on 8 October 2024, confirmed further progression, with both new and enlarging hepatic lesions compared with the 21 August scan. In December 2024, the patient presented to our emergency department with nausea and vomiting and was diagnosed with a perforated gastric ulcer. He successfully underwent gastric perforation repair and received supportive care, with subsequent clinical improvement and discharge. The patient was later lost to follow-up.

## 3 Discussion

Hidradenocarcinoma is a rare and aggressive malignancy with limited treatment options. We report a patient who developed repeated local recurrence and distant metastases despite multiple surgical excisions. The patient was treated with sequential immunochemotherapy combined with radiotherapy. The first- and second-line regimens achieved progression-free survival (PFS) of 6 and 8 months, respectively, without significant immune-related adverse events. This case underscores the potential role of immunotherapy in managing this challenging disease and highlights the importance of a multidisciplinary approach to optimize outcomes.

Accurate histopathological classification is fundamental for guiding patient management and facilitating the sharing of clinical experiences. However, carcinomas derived from sweat glands exhibit heterogeneous and complex histopathologic features, leading to varied classifications and nomenclature across studies without a clear consensus [7,15]. According to the International Agency for Research on Cancer (IARC)/World Health Organization (WHO) Classification of Skin Tumors (5th edition), malignancies with apocrine or eccrine differentiation represent one of the four major groups of cutaneous adnexal carcinomas and include more than 15 entities, such as hidradenocarcinoma, porocarcinoma, apocrine carcinoma, microcystic adnexal carcinoma, and adenoid cystic carcinoma [16]. These subtypes differ substantially in pathogenesis, clinical behavior, and prognosis.

Regardless of subtype, surgery remains the preferred treatment when feasible [16]. Surgical approaches include wide local excision and Mohs micrographic surgery, which are performed in more than 90% of cases [2,17]. Despite the high rate of lymph node metastasis, the benefit of regional lymph node dissection remains controversial. Some studies suggest that sentinel lymph node biopsy may help guide treatment decisions and predict prognosis [8,18]. Therapeutic challenges arise when surgery is not feasible or when recurrence occurs after resection [9]. Adjuvant radiotherapy may be considered for patients at high risk of recurrence, such as those with high locoregional disease burden, extensive lymph node involvement, or tumors arising in the head and neck [16]. Radiotherapy also serves as an alternative definitive treatment for unresectable disease and as a palliative option for metastatic disease [19,20].

Various chemotherapy regimens have been reported, including combinations of 5-fluorouracil with platinum-based agents [21], paclitaxel with carboplatin [22], docetaxel [23], and capecitabine with carboplatin [24]. However, responses have been inconsistent and generally limited. More recently, electrochemotherapy—combining low-dose chemotherapy with electroporation to enhance drug uptake—has emerged as a potential treatment option for skin tumors [9].

Targeted therapy may complement conventional treatments. Immunohistochemical analysis of primary and metastatic lesions from 14 patients with metastatic adnexal carcinoma demonstrated frequent epidermal growth factor receptor (EGFR) overexpression [25]. Consistent with this finding, a patient with metastatic porocarcinoma and strong EGFR expression achieved a six-month complete response with paclitaxel, cetuximab, and radiotherapy before disease progression [26]. For HER2-targeted therapy, a reported case of metastatic hidradenocarcinoma with *HER2* amplification achieved SD with trastuzumab and radiotherapy [27]. In contrast, our patient had low HER2 expression (1+), and in the absence of established criteria supporting anti-HER2 therapy in hidradenocarcinoma, immunotherapy was prioritized. Furthermore, genomic alterations in genes such as *PIK3CA*, *TP53*, *ALPK1*, *BRCA*, and *FGFR2* have been identified across sweat gland carcinomas (SGCs) subtypes, suggesting potential roles for inhibitors targeting the Akt/PI3K/mTOR and NF-κB pathways in suppressing tumor growth [25,28,29,30]. Nevertheless, evidence-based molecular targets for routine clinical use remain lacking.

Given the success of immunotherapy in malignancies such as melanoma, breast cancer, and sebaceous carcinoma [31], its role in SGCs has gained interest. Studies have shown heterogeneous PD-L1 expression and CD8^+^ tumor-infiltrating lymphocytes (TILs) in these tumors, with generally higher levels observed in apocrine carcinoma and invasive EMPD [32], suggesting potential sensitivity to ICIs. Conversely, a retrospective analysis of hidradenocarcinoma reported a predominantly non-inflamed tumor microenvironment [33]. Obermann et al. [34] reported the first case of metastatic hidradenocarcinoma with a marked response to the anti-programmed death-1 (PD-1) antibody nivolumab. Immunohistochemical analysis demonstrated an increase in CD3^+^, CD4^+^, and CD8^+^ lymphocyte infiltration after two weeks of treatment. However, population-based evidence supporting immunotherapy in SGCs remains limited. Additional case reports evaluating immunotherapy in these tumors are summarized in Table 1.

**Table 1:** Case reports of immunotherapy in sweat gland carcinoma.

Study	Age/Sex	SGC Type	PD-L1 Expression	PD-L1 Testing Method	MSS	Gene Alteration	Previous Treatment	Line	ICIs Regimen	Outcomes
Rogatsch 2018 [35]	83/Male	apocrine carcinoma	60%	Tumor biopsy (28-8 clone)	Unknown	Unknown	None	1	Pembrolizumab	PR, PFS of >12 months
Lee 2019 [24]	67/Female	porocarcinoma	Numerous foci of positivity	Tumor biopsy	Unknown	Unknown	Carboplatin + capecitabine	2	Pembrolizumab	PR, PFS of >25 months
Gupta 2020 [22]	75/Female	eccrine carcinoma	Unknown	Not performed	Unknown	*NOTCH*	Paclitaxel + capecitabine	2	Pembrolizumab	PD
Comito 2021 [36]	70/Male	porocarcinoma	5%	Tumor biopsy	Unknown	None	None	1	Pembrolizumab	PD
Obermann 2021 [34]	71/Female	hidradenocarcinoma	50%	Tumor biopsy	MSI-H	*TP53, NOTCH*, etc., TMB 186/Mb	None	1	Nivolumab	CR
Singh 2021 [37]	79/Male	porocarcinoma	Negative	Tumor biopsy (ZR3 immunostain)	Unknown	Unknown	None	1	Pembrolizumab	PR, PFS of >27 months
Dai 2023 [38]	55/Female	sweat gland carcinoma	10%	Blood test	Unknown	Unknown	Docetaxel + nedaplatin	2	Camrelizumab + paclitaxel + nedaplatin	PD
Patel 2024 [39]	50/Unknown	cutaneous adnexal carcinoma	30%	Tumor biopsy (22C3 clone)	Stable	*TP53*, TMB 3/Mb	None	1	Pembrolizumab	SD, PFS of >10 months

Abbreviations: CR, complete response; ICIs, immune checkpoint inhibitors; MSI, microsatellite instability; MSS, microsatellite stability; PD, progressive disease; PFS, progression-free survival; PR, partial response; SGC, sweat gland carcinoma; SD, stable disease; TMB, tumor mutational burden.

Comparison with previously reported cases shows that most patients received single-agent PD-1 inhibitors, whereas our patient underwent sequential immunochemotherapy using two different PD-1 inhibitors combined with an anti-angiogenic tyrosine kinase inhibitor (TKI). PD-L1 expression among responders ranged from focal staining to 60%, yet our patient achieved durable disease control despite low PD-L1 (CPS = 5). Notably, PD-L1 status was unavailable in several published cases, limiting cross-study comparisons and underscoring the need for cautious interpretation. These findings suggest that, similar to melanoma and other skin cancers, low PD-L1 expression may not preclude benefit from immunotherapy SGCs, and that combination strategies merit further investigation. However, the literature on SGCs encompasses biologically diverse malignancies with differing pathogenesis and treatment response. Therefore, conclusions drawn from one subtype may not be directly applicable to another.

Our therapeutic strategy was guided by institutional experience and treatment approaches used for other aggressive cutaneous malignancies. First-line treatment consisted of sintilimab combined with cisplatin and fluorouracil, selected based on reported activity in cutaneous squamous cell carcinoma and other adnexal tumors [40,41]. Following disease progression during maintenance sintilimab therapy, second-line treatment was initiated with the goals of improving systemic disease control, addressing potential treatment resistance, and maintaining tolerability in the setting of widespread metastases. Informed by our institutional experience and studies in aggressive skin cancers such as melanoma [42,43], the patient received toripalimab combined with nab-paclitaxel and the anti-angiogenic agent anlotinib. This approach was partly based on the hypothesis that resistance to one PD-1 inhibitor may not result in complete cross-resistance to another, given potential differences in epitope recognition and immune effects. Although both sintilimab and toripalimab target PD-1, they bind distinct epitopes within the FG loop [44,45,46,47,48]. However, whether these structural differences translate into clinically meaningful activity remains unclear, and most established resistance mechanisms to PD-1 blockade—such as mutations in JAK1/2 or B2M [49,50], and upregulation of alternative immune checkpoints including LAG-3 and TIM-3 [51,52]—are unlikely to be overcome by switching antibodies alone. Therefore, the observed clinical benefit cannot be attributed to sequential PD-1 inhibition alone and was likely multifactorial, including modification of the chemotherapy backbone and the addition of anlotinib.

The addition of anlotinib was based on prior evidence suggesting that anti-angiogenic agents may enhance treatment efficacy by normalizing tumor vasculature, improving immune cell infiltration, and facilitating drug delivery [53,54,55,56]. Supporting this strategy, the phase II CAP-03 trial demonstrated promising activity of camrelizumab combined with apatinib and temozolomide in advanced acral melanoma [57], and the phase II LEAP-004 study showed clinically meaningful responses with lenvatinib plus pembrolizumab after prior PD-1 therapy [58]. Although the phase III LEAP-003 trial did not demonstrate a survival benefit in the first-line setting [59], these findings collectively suggest that anti-angiogenic agents may enhance immunotherapy activity in selected contexts. In addition, the development of liver metastases in our patient further supported the use of anti-angiogenic therapy, as previous studies have suggested potential activity of these agents in highly vascular metastatic sites such as the liver [60,61]. Taken together, the responses observed with both treatment lines suggest that multimodal combination strategies, rather than sequential PD-1 inhibition alone, may represent a reasonable exploratory approach for this treatment-resistant malignancy.

Notably, our patient derived sustained clinical benefit despite biomarkers typically associated with limited immunotherapy response, including low PD-L1 expression (CPS = 5) and MSS status. Genomic profiling identified mutations in *BCL2L11* and *FOXA1*, which may provide potential biological explanations. *BCL2L11,* also known as BIM, functions downstream of PD-1 signaling and regulates apoptosis in effector CD8^+^ T cells [62], while *FOXA1* has been reported to modulate PD-L1 expression and suppress interferon signaling and antigen presentation [63,64]. In melanoma, elevated BIM protein levels in circulating tumor-reactive CD8^+^ T cells have been associated with improved response to anti-PD-1 therapy [62]. Additionally, preclinical studies have shown that FOXA1 knockdown enhances CD8^+^ T-cell infiltration and increases sensitivity to PD-1 blockade [64]. These findings raise the possibility that alterations in *BCL2L11* and *FOXA1* may have contributed to immunotherapy response in our patient despite unfavorable biomarkers. However, these interpretations remain speculative, as direct clinical evidence linking these mutations to immunotherapy response is currently lacking.

A major strength of this report is the detailed longitudinal documentation of sequential immunochemotherapy combined with anti-angiogenic therapy, together with molecular profiling and radiologic follow-up in a rare malignancy. We acknowledge several important limitations. Additional key immunologic biomarkers, such as TMB, detailed TIL subsets (e.g., CD4^+^ and CD8^+^ T cells), and immune gene expression profiling, were not assessed because these tests were not part of routine clinical practice and no residual tissue was available for retrospective analysis. The potential roles of *BCL2L11* and *FOXA1* mutations remain speculative due to the lack of direct experimental or clinical evidence linking these alterations to immunotherapy response. Furthermore, although our patient achieved sustained disease control, the best overall response was stable disease, which limits the strength of conclusions regarding treatment efficacy. Prospective studies incorporating comprehensive immune profiling are needed to better understand the immunobiology of hidradenocarcinoma and to identify predictive biomarkers beyond PD-L1 and microsatellite instability (MSI) status. Further functional studies and validation in larger cohorts are also required to determine whether *BCL2L11* or *FOXA1* mutations influence immunotherapy response and may serve as predictive biomarkers in hidradenocarcinoma and other rare malignancies.

## 4 Conclusion

In conclusion, this case report suggests that sequential immunochemotherapy combined with an anti-angiogenic TKI may provide clinically meaningful disease control in metastatic hidradenocarcinoma. The observed response to PD-1 blockade-based regimens, despite low PD-L1 expression and MSS, supports a potential role for immunotherapy in this aggressive malignancy. The addition of anlotinib may have further enhanced treatment efficacy through vascular normalization and improved drug delivery. Although this report adds to the limited evidence supporting PD-1 inhibition in hidradenocarcinoma, these findings require validation in larger, multicenter studies. Further research is needed to establish standardized treatment strategies and to identify biomarkers predictive of response.

## Data Availability

The authors confirm that the data supporting the findings of this study are available within the article and its Supplementary Materials.

## Supplementary Materials

The supplementary material is available online at https://www.techscience.com/doi/10.32604/or.2026.080462/s1.

## Abbreviations

AR	androgen receptor
CPS	combined positive score
CR	complete response
CT	computed tomography
CTCAE	common terminology criteria for adverse events
EGFR	epidermal growth factor receptor
EMPD	extramammary Paget disease
GCDFP-15	gross cystic disease fluid protein 15
H&E	hematoxylin and eosin
HER2	human epidermal growth factor receptor 2
IARC	international agency for research on cancer
ICI	immune checkpoint inhibitor
IV	intravenously
MRI	magnetic resonance imaging
MSI	microsatellite instability
MSS	microsatellite stable
PD	progressive disease
PD-(L)1	programmed death-(ligand) 1
PET-CT	positron emission tomography–computed tomography
PFS	progression-free survival
PR	partial response
RECIST	response evaluation criteria in solid tumors
SD	stable disease
SGC	sweat gland carcinoma
TKI	tyrosine kinase inhibitor
TMB	tumor mutational burden
WHO	World Health Organization
