# Supplementary material for: Stable Disease Achieved with Sequential Immunochemotherapy and Anti-Angiogenic TKI in Recurrent Metastatic Hidradenocarcinoma: A Case Report and Literature Review
Source: Oncol Res. 2026 Jul 16;34(8):30. doi: 10.32604/or.2026.080462 (PMC13397351; doi:10.32604/or.2026.080462)
Supplement: Supplementary file 1 [file OncolRes-34-80462-s001.zip › TSP_OR_80462-s001.docx]

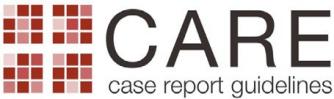

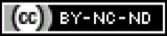

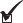
**CARE Checklist of information to include when writing a case report**

| **Topic** | **Item No** | **Checklist item description** | **Reported on Page Number/Line Number** | **Reported on Section/Paragraph** |
| --- | --- | --- | --- | --- |
| Title | 1 | The diagnosis or intervention of primary focus followed by the words “case report” | 1/3-5 | Front Matter/Title |
| Key Words | 2 | 2 to 5 key words that identify diagnoses or interventions in this case report, including "case report" | 1/38-39 | Front Matter/Key words |
| Abstract  (Structured summary) | 3a | Background: state what is known and unknown; why the case report is unique and what it adds to existing literature. | 1/21-24 | Abstract/Paragraph 1 |
|  | 3b | Case Description: describe the patient’s demographic details, main symptoms, history, important clinical findings, the main diagnosis, interventions, outcomes and follow-ups. | 1/25-31 | Abstract/Paragraph 2 |
|  | 3c | Conclusions: summarize the main take-away lesson, clinical impact and potential implications. | 1/32-37 | Abstract/Paragraph 3 |
| Introduction | 4 | One or two paragraphs summarizing why this case is unique **(may include references)** | 2/41-77 | Introduction/Paragraph 1-5 |
| Patient Information | 5a | De-identified patient specific information | 2/80 | Case Presentation/Paragraph 1 |
|  | 5b | Primary concerns and symptoms of the patient | 2/81-82 | Case Presentation/Paragraph 1 |
|  | 5c | Medical, family, and psycho-social history including relevant genetic information | 2/80 | Case Presentation/Paragraph 1 |
|  | 5d | Relevant past interventions with outcomes | 2/82-89 | Case Presentation/Paragraph 1 |
| Clinical Findings | 6 | Describe significant physical examination (PE) and important clinical findings | 2-3/81-89 | Case Presentation/Paragraph 1 |
| Timeline | 7 | Historical and current information from this episode of care organized as a timeline | 5/154 | Case Presentation/Fig 2 |
| Diagnostic Assessment | 8a | Diagnostic testing (such as PE, laboratory testing, imaging, surveys). | 2-3/83-101 | Case Presentation/Paragraph 1 |
|  | 8b | Diagnostic challenges (such as access to testing, financial, or cultural) | 2-3/82-101 | Case Presentation/Paragraph 1 |
|  | 8c | Diagnosis (including other diagnoses considered) | 4/112 | Case Presentation/Paragraph 2 |
|  | 8d | Prognosis (such as staging in oncology) where applicable | 4/112 | Case Presentation/Paragraph 2 |
| Therapeutic Intervention | 9a | Types of therapeutic intervention (such as pharmacologic, surgical, preventive, self-care) | 4/113-114 | Case Presentation/Paragraph 2 |
|  | 9b | Administration of therapeutic intervention (such as dosage, strength, duration) | 4/114-120 | Case Presentation/Paragraph 2 |
|  | 9c | Changes in therapeutic intervention (with rationale) | 4/128-130, 4/143-145, 4/147-148 | Case Presentation/Paragraph 3-4 |

| Follow-up and Outcomes | 10a | Clinician and patient-assessed outcomes (if available) | 4/120, 4/122-123, 4/131-136, 4/145-147 | Case Presentation/Paragraph 2-4 |
| --- | --- | --- | --- | --- |
|  | 10b | Important follow-up diagnostic and other test results | 4/125-128 | Case Presentation/Paragraph 2-3 |
|  | 10c | Intervention adherence and tolerability (How was this assessed?) | 4/120-122, 4/136-142 | Case Presentation/Paragraph 2-3 |
|  | 10d | Adverse and unanticipated events | 4/137-142, 4/150-152 | Case Presentation/Paragraph 3-4 |
| Discussion | 11a | A scientific discussion of the strengths AND limitations associated with this case report | 5/168-170, 8/273-286 | Discussion/Paragraph 1, 11 |
|  | 11b | Discussion of the relevant medical literature **with references** | 5-8/171-272 | Discussion/Paragraph 2-10 |
|  | 11c | The scientific rationale for any conclusions (including assessment of possible causes) | 8/261-272 | Discussion/Paragraph 10 |
|  | 11d | The primary “take-away” lessons of this case report (without references) in a one paragraph conclusion | 9/287-295 | Conclusion/Paragraph 1 |
| Patient Perspective | 12 | The patient should share their perspective in one to two paragraphs on the treatment(s) they received | 3/112-114 | Case Presentation/Paragraph 2 |
| Informed Consent | 13 | Did the patient give informed consent? Please provide if requested | **Yes √** | **No** |

*As the checklist was provided upon initial submission, the page number/line number reported may be changed due to copyediting and may not be referable in the published version. In this case, the section/paragraph may be used as an alternative reference.
